# Effect of flavonoids on antimicrobial activity of microorganisms present in dental plaque

**DOI:** 10.1016/j.heliyon.2019.e03013

**Published:** 2019-12-13

**Authors:** Gloria Gutiérrez-Venegas, Juan Arturo Gómez-Mora, Marco Antonio Meraz-Rodríguez, Mónica Arisbet Flores-Sánchez, Laura Fabiola Ortiz-Miranda

**Affiliations:** Laboratorio de Bioquímica, División de Estudios de Posgrado e Investigación, Facultad de Odontología, Universidad Nacional Autónoma de México, Ciudad de México, 04510, Mexico

**Keywords:** Biochemistry, Microbiology, Pharmaceutical science, Pharmacology, Flavonoids, Dental plaque, Caries

## Abstract

**Purpose:**

Dental caries is a multi-factorial oral disease, requiring a susceptible host, cariogenic microorganisms and suitable substrate. Caries is extended worldwide in spite of the availability of countless prophylactic means, including fluoride toothpaste and dental sealers. Many efforts have been made to achieve isolation of pure natural products for medicinal use. Flavonoids are bioactive polyphenol compounds possessing multidimensional effects such as antibacterial action.

**Methods:**

The present study targeted the characterization of antibacterial and antifungal activity of various flavonoids (apigenin, catechin, luteolin, morin, myricetin, naringin, quercetin and rutin). Nine strains present in dental plaque were used: *Agreggatibacter actinomycetemcomitans, Actinomyces naeslundii, Actinomyces viscosus, Enterococcus faecalis, Escherichia coli, Lactobacillus casei, Staphylococcus aureus, Streptococcus oralis* and *Streptococcus sanguinis* as well as *Candida albicans* fungal strain.

**Results:**

Results revealed that luteolin, morin, naringin, quercetin and rutin effectively inhibited bacterial and fungal growth. However, morin was the most effective flavonoid.

**Conclusion:**

It might then be concluded that flavonoids show bacteriostatic effect on all of tested bacteria and fungus.

## 1 Introduction

Dental plaque is a microbial community that colonizes the surfaces of the teeth in a structurally and organized biofilm [1, 2]. Microbial homeostasis remains stable over time. However, factors such as diet or immune response disrupt biofilm stability and promote diseases such as caries [3, 4, 5]. Predominant species in diseased sites are different from those found in healthy sites [6, 7]. Several studies have identified Gram-positive bacteria such as streptococci as a most important colonizer of early enamel biofilm. *Streptococcus* conforms 63% of bacteria isolated after 4 h of plaque formation, communities that were dominated by *Streptococcus oralis* and *mitis* strains conform 86% of bacteria isolated after 8 h [7, 8, 9]. A variety of other bacteria such as *Actinomyces* was reported to be present, followed by *Lactobacillus* [10]. Prevalence of *Staphylococcus aureus* in the oral cavity and its role in periodontal infection it is as yet to be demonstrated. However, it has been isolated in suppurative parotitis, angular chelitis and dentoalveolar infections [11]. *Enterococcus faecalis* is a Gram-positive facultative coccus that may opportunistically colonize periodontal pockets and contribute to periodontal breakdown in heavily infected sites [12].

Microorganisms present in the oral cavity interact using their metabolic products or exchanging molecular signals. *Candida albicans* has been shown to be the predominant pathogen; it is an innocuous commensal of microbial communities in human oral cavity [13]. Its primary location is the posterior side of the tongue and other oral sites such as the mucosa, and acts as a secondary colonizer in the film that covers dental surfaces [13]. When *Candida* becomes virulent it generates candidiasis, which can be manifested through various clinical forms, involving one or more oral sites followed by total mouth affectation [14].

In order to reduce dental caries, numerous studies have been conducted. Dental therapy is primarily targeted through the prevention of *Streptococcus* colonization by focusing on removing the whole dental plaque [15]; this procedure will remove both commensal bacteria along with oral pathogens. Nevertheless, it creates niches for pathogens to repopulate the oral cavity [15]. Some other studies were conducted to inhibit adherence with antagonists such as sucrose analogues or through antibiotic usage [16]. Ecological disruptions therefore result in negative secondary infections. To address these effects alternative research has been conducted with natural products possessing anti-bacterial activity [17].

Flavonoids, a family of polyphenolic compounds, widely distributed in the plant kingdom are consumed in significant amounts as part of the human diet [18]. Different studies have shown that flavonoids in a healthy diet have potentially beneficial effects as antimicrobial agents. Polyphenols as catechin play a role on different bacterial strains belonging to different species such as *Escherichia coli, Staphylococcus aureus, Streptococcus mutans, Streptococcus sanguinis* and *Actinomyces viscosus* [19, 20, 21].

Recently, some research groups have identified two potential anti-caries agents found in propolis, a natural bioactive product: apigenin (4′,5,7,-trihydroxyflavone) a non-toxic dietary flavonoid found in fruits and vegetables. It displays activity against streptococcal membranes by increasing their proton permeability as well as inhibiting acid production by *Streptococcus mutans* within biofilms [22, 23]. Luteolin (3′,4′,5,7-tetrahydoxyflavone) is a flavonoid possessing a variety of pharmacological activities, including antioxidant, anti-inflammatory and anticarcinogenic functions [24]. Myricetin (3,5,7,3′,4′,5’ hexahydroxylfavone) largely distributed in berries, showed activity against *Porphyromonas gingivali*s with MIC values in the range of 62.5–125 μg/mL [25]. Morin (2-(2,4-dihydroxiphenyl)-3,5,7-trihydroxychromen-4-one), showed inhibitory activity against *Salmonella enteritidis, Escherichia coli, Bacillus subtilis* and *Staphylococcus aureus* [26, 27]. Difficulties in treatment of resistant microbes serve as a challenge to discover new drugs that can be effectively used against different resistant microorganisms [28]. Since plant metabolites are not commonly used, they can be considered as a therapy against different microorganisms. In view of the fact that flavonoids represent an emerging threat of dental bacteria, this study was designed to evaluate the effect of selected single flavonoids (apigenin, catechin, luteolin, morin, myricetin, naringin, quercetin and rutin) for their activities against dental film bacteria and *Candida albicans* to prevent Candidiasis.

## 2 Materials and methods

### 2.1 Materials

**Figure 1:**
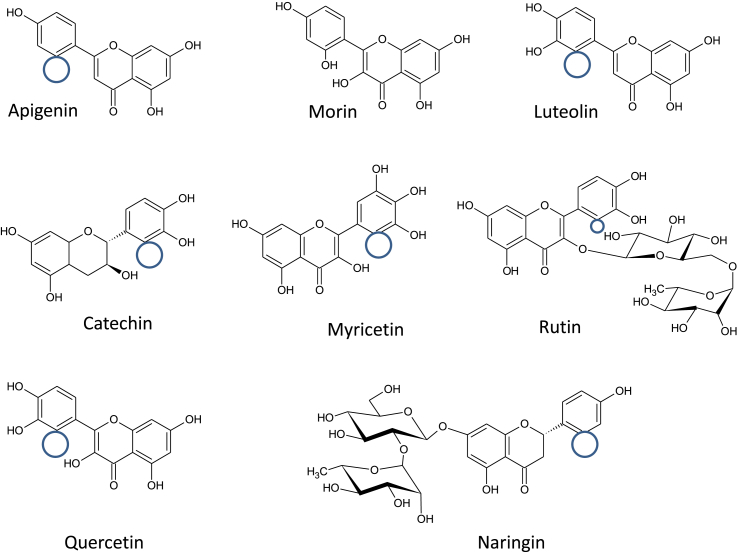
Structures of different flavonoids when compared to morin. Morin possesses a hydroxyl group placed at R6′ that the other flavonoids lack in their structure. Molecules contain hydroxyl group substitutions at R3, R7 and R4’. Apigenin, luteolin and naringin lack the hydroxyl group at R3. Catechin, luteolin, myricetin, quercetin and rutin possess one hydroxyl group at R5′, which is absent in morin.

Apigenin, catechin, morin, myricetin, luteolin, naringin, quercetin and rutin all within a purity range of <95% were obtained from Sigma-Aldrich (St. Louis Mo, USA). The structures of the flavonoids used in this study are shown in Figure 1 and Table 1. Potassium tellurite, barium chloride, pentone crystal violet, Lugol's iodine, safranine, alcohol, and hydrogen peroxide were purchased from DIFCO (1 Becton Drive Franklin Lakes, NJ 07417 United States).

**Table 1:** Flavonoids used in this study.

Flavonoids	Classification	IUPAC Name
Apigenin	Flavone	5,7-Dihydroxy-2-(4-hydroxyphenyl)-4H-1-benzopyran-4-one
Luteolin	Flavone	2-(3,4-dihydroxyphenyl)-5,7-dihydroxychromen-4-one
Catechin	Flavonol	(2R,3S)-2-(3,4-dihydroxyphenyl)-3,4-dihydro-2H-chromene-3,5,7-triol
Morin	Flavonol	2-(2,4-dihydroxyphenyl)-3,5,7-trihydroxychromen-4-one
Myricetin	Flavonol	3,5,7-trihydroxy-2-(3,4,5-trihydroxyphenyl)chromen-4-one
Quercetin	Flavonol	2-(3,4-dihydroxyphenyl)-3,5,7-trihydroxychromen-4-one
Rutin	Flavonol	2-(3,4-dihydroxyphenyl)-5,7-dihydroxy-3-[(2S,3R,4S,5S,6R)-3,4,5-trihydroxy-6-[[(2R,3R,4R,5R,6S)-3,4,5-trihydroxy-6-methyloxan-2-yl]oxymethyl]oxan-2-yl]oxychromen-4-one
Naringin	Flavanone	7-2-O-(6-Deoxi-α-L-manopiranosil)-β-D-glucopiranosiloxi-2,3-dihidro-5-hidroxi-2-(4-hidroxifenil)-4H-1-benzopyran-4-one

### 2.2 Media preparation

***Candida albicans*** (ATCC 10231, Manassas, VA, USA) was grown in Sabouraud agar (DIFCO, St Louis, Mo) for 24 h at 37 °C. Identity was confirmed by conventional identification methods, such as germ tube induction at 37 °C, microscopic morphology and chlamydospore formation in corn meal agar (Oxoid, Basingstoke, United Kingdom). ***Actinomyces naeslundii*** (ATCC 12104) and ***Actinomyces viscosus*** (ATCC 15987) were grown in Tryptic Soy Agar broth containing 1 μg/mL of yeast extract, 5 μg/mL of hemin and 1 μg/mL of menadione in an anaerobic system in atmosphere of 80% N_2_, 10% CO_2_ and 10% H_2_ at 35 °C for 16 h. Blood Agar (DIFCO, St Louis, Mo) adding 5 mL of hemin (0.05% concentration) and 5 mL of Vitamin K stock (0.005% concentration) at anaerobic conditions and 37 °C for 48 h. ***Lactobacillus casei*** (ATCC 393) was grown in Man, Rogosa and Sharpe (MRS) broth agar (Oxoid, Basingstoke, England) and incubated at 37 °C for 16 h. ***Enterococcus faecalis*** *(ATCC* 8043) was grown in Trypticase Soy Broth (Becton Dickinson Microbiology Systems, Cockeysville, MD, USA), 1 μg/mL yeast extract and 15 g/L agar (Difco Laboratories, Detroit, MI, USA) and incubated at 37 °C in a reduced O_2_ atmosphere. Blood Agar (DIFCO, St Louis, Mo) adding 5 mL of hemin (0.05% concentration) and 5 mL of Vitamin K stock (0.005% concentration) at anaerobic conditions and 37 °C for 24 h. ***Escherichia coli*** (ATCC 25922) was grown in Luria Bertani Miller broth containing 1% NaCl, 1% peptone and 0.5% yeast extract. ***Streptococcus sanguinis*** (ATCC 10556) and ***Streptococcus oralis*** (ATCC 35037) were grown in Trypticase Soy Agar broth with defibrinated sheep blood at 37 °C in 5% CO_2_ atmosphere. Blood Agar (DIFCO, St Louis, Mo) adding 5 mL of hemin (0.05% concentration) and 5 mL of Vitamin K stock (0.005% concentration) at anaerobic conditions and 37 °C for 48 h. ***Agreggatibacter actinomycetemcomitans*** (ATCC 43718) was grown in Blood Agar (DIFCO, St Louis, Mo) adding 5 mL of hemin (0.05% concentration) and 5 mL of Vitamin K stock (0.005% concentration) at anaerobic conditions and 37 °C for 48 h. ***Staphylococcus aureus*** (ATCC 25923) was grown in Blood Agar (DIFCO, St Louis, Mo) adding 5 mL of hemin (0.05% concentration) and 5 mL of Vitamin K stock (0.005% concentration) at 37 °C.

All strains were stored at -80 °C in the medium with 20% glycerol (v/v). Prior to use, the aliquots of these cultures were reactivated in their medium incubated at 37 °C for 24 h. All mediums were prepared according Manufacturer's instructions.

### 2.3 Preparation of 0.5 Mc Farland standards

One mL of concentrated sulfuric acid (H_2_SO_4_) was added to 99 mL of distilled water and mixed thoroughly. After this, 1% v/v of barium chloride (BaCl_2_) solution was achieved by mixing 1.0 g in 100 mL of distilled water. Concentrated H_2_SO_4_ solution was mixed with the barium chloride solution to produce a turbid solution of barium sulfate (BaSO_4_). After that, a small volume of the turbid solution was transferred into a capped tube, of the same type as that used for test and control inocula, after this it was stored in a well-sealed container at temperatures between 20-28 °C [29].

### 2.4 Gram Staining

Gram Staining was carried out as a control on all bacteria. A drop of saline solution was used to solubilize an aliquot of bacterial colonies on a clean glass slide to achieve a thin, uniform smear. This was then allowed to air-dry, after which the smear was heat-fixed over a Bunsen flame and allowed to cool. Crystal violet was added to cover the smear for 1 min, after which it was washed with tap water. Lugol's iodine was added to this preparation and left for 1 min; to then be washed off. Discoloration was achieved with acid alcohol, quickly and uniformly added to the slide and left for about 5 s, after which it was rapidly washed off with water. Counter-staining was conducted for 1 min by addition of safranine and then washed with water and air-dried.

### 2.5 Preparation of flavonoid-impregnated discs

Whatman number 1 filter papers were used to prepare 5 mm diameter discs. Discs were autoclave-sterilized and then dried during autoclave cycle. Discs were impregnated with flavonoid (<97%) (SIGMA) dissolved in dimethylsulfoxide, which was used as a vehicle. And Ampiciline 0.1 μg/mL as a positive control.

### 2.6 Disc diffusion method

Disc diffusion method for antimicrobial susceptibility test was carried out according to the standard method designed by Kirby-Bauer to evaluate the presence of antimicrobial activities of flavonoids. A bacterial suspension adjusted to 0.5 McFarland standard (1.5 × 10^8^ CFU/mL) was used to inoculate Blood Agar + Hemin + Vitamin K, Sabouraud, LB and BHI (according to the bacteria/fungi) plates evenly using a sterile swab. Flavonoid-impregnated discs were individually placed on the Blood Agar, Sabouraud, LB or BHI surface with flamed forceps and gently pressed down to ensure contact with the agar surface. Discs were impregnated with flavonoids, at increasing doses between 0.1 mg/mL and 1.0 mg/mL every 0.1 units and 5.0 mg/mL. Five discs per plate were placed in order to avoid both reflection waves from the edges of the petri dishes and overlapping inhibition rings. The plates were then incubated at 37 °C for around 18 h in inverted position to look for zones of inhibition. And some in anaerobic conditions. As soon as we observed growth inhibition of bacteria, the stocks were diluted in order to get exact doses of flavonoid.

### 2.7 Statistical analysis

To ensure validity of statistical analyses all experiments were performed in triplicate. Results were expressed as mean ± SD**.** One-way ANOVA with multiple comparisons was performed on the data sets generated by using Predictive Analysis Software. Differences for *ρ* values <0.05–0.01 were considered significant. Also, Tukey test was performed for post-hoc test in order to make all of the possible comparisons between flavonoids per bacteria.

## 3 Results

### 3.1 Effect of flavonoids activities against *Actinomyces naeslundii* and *Actinomyces viscosus*

We found that rutin, quercetin and morin showed antibacterial activity against *A. naeslundii* and *A. viscosus*. Rutin was found to be the most effective. Indeed, the concentration at which rutin showed activity against studied bacteria was 0.61 mg/mL (1 ± 0.1 cm). Luteolin only had impact at the 5 mg/mL (1.7 × 10^−2^M) concentration for *A. viscosus* while Morin at 10 mg/mL (3.3 × 10^−2^ M) for both strains. Also, Naringin had an impact only on *A. naeslundii* at 10 mg/mL concentration (1.7 × 10^−2^ M) and 30 mg/mL (5.1 × 10^−2^M). Finally, quercetin showed an activity at 10 mg/mL (3.3 × 10^−2^ M) and 30 mg/mL (9.9 × 10^−2^M) for both strains. In the case of ^b^*A.n.* there are significant differences between rutin, quercetin, naringin and morin (*ρ* < 0.05), and for ^c^*A.v.* there are significant differences between luteolin, morin and rutin (*ρ* < 0.05).

No additional inhibitory effect was detected at 1 mM doses. However, apigenin, catechin and myricetin were found to be exerting no impact on both species of tested *Actinomyces*, and luteolin for *A. naeslundii* (Table 2).

**Table 2:** Antimicrobial effect of flavonoids in different strains of Bacteria.

Bacteria	Apigenin	Catechin	Luteolin	Morin	Myricetin	Naringin	Quercetin	Rutin
*Aggregatibacter actinomycetemcomitans*	0.00	0.86 ± 0.17^a^*	0.73 ± 0.02^a^*	0.75 ± 0.01*	0.00	0.73 ± 0.009^a^*	0.81 ± 0.05	0.00
*Actinomyces naeslundii*	0.00	0.00	0.00	0.69 ± 0.03^b^*	0.00	0.74 ± 0.007^b^*	0.74 ± 0.009^b^*	1.03 ± 0.03^b^*
*Actynomyces viscosus*	0.00	0.00	0.75 ± 0.03^c^*	0.79 ± 0.04^c^*	0.00	0.00	0.83 ± 0.003	0.97 ± 0.09^c^*
*Candida albicans*	0.00	0.00	0.66 ± 0.02^d^*	0.81 ± 0.05	0.00	1.4 ± 0.11^d^*	0.84 ± 0.01	0.73 ± 0.06^d^*
*Escherichia coli*	0.00	0.00	0.83 ± 0.03	0.74 ± 0.02	0.00	0.77 ± 0.01	0.84 ± 0.07	0.87 ± 0.12
*Enterococcus faecalis*	1.30 ± 0.45^e^*	0.00	0.97 ± 0.12	1.01 ± 0.18	0.00	0.87 ± 0.08^e^*	0.00	0.93 ± 0.03
*Lactobacillus casei*	0.00	0.00	0.00	1.03 ± 0.03	0.00	1.10 ± 0.06^f^*	0.00	0.9 ± 0.06^f^*
*Staphylococcus aureus*	1.17 ± 0.03^g^*	1.39 ± 0.28^g^*	0.81 ± 0.04^g^*	1.03 ± 0.03	0.00	0.87 ± 0.036	0.97 ± 0.14	0.65 ± 0.04^g^*
*Streptococcus oralis*	0.00	0.00	0.00	0.00	0.00	0.00	0.00	0.00
*Streptococcus sanguinis*	0.00	0.00	0.00	0.00	0.00	0.00	0.00	0.00

Results are expressed as means ± SD (n = 3). For each column, values were statistically significant difference * = (p < 0.05) vs *a* to *g* = most active flavonoid, determinated using ANOVA apply for different flavonoids in same Bacteria.

### 3.2 Effect of flavonids activities against *Enterococcus faecalis*

Apigenin, rutin, luteolin, naringin and morin showed some activities against *Enterococus faecalis*. However, catechin, myricetin and quercetin were found inactive against this bacterium (Table 2). For ^**e**^*E.f.* there are significant differences between apigenin and naringin (*ρ* < 0.05).

### 3.3 Effect of flavonoids against *Escherichia coli*

To compare flavonoids antibacterial activity, they were tested for ability to limit *Escherichia coli* growth. We found that rutin, luteolin and naringin at 100 μM doses inhibited the growth of Gram-negative bacteria strain, *Escherichia coli*. Rutin exhibited inhibition to a higher concentration of 1 mM. It was also found that at higher concentrations they also showed inhibitions from naringin at 30 mg/mL (5.1 × 10^−2^ M). Morin 10 mg/mL (3.3 × 10^−2^ M) and quercetin at 10 mg/mL (3.3 × 10^−2^ M) (Table 2).

### 3.4 Effect of flavonoids against *Lactobacillus casei*

The antibacterial activity was analyzed through paper disk method using *Lactobacillus casei*. Rutin and naringin each showed antibacterial activity at 100 μM, but morin was active at higher doses of 1 mM (Table 2). For ^**f**^*L.c*. we can observe significant differences between naringin and rutin (*ρ* < 0.05).

### 3.5 Effect of flavonoids against *Staphylococcus aureus*

The antimicrobial activity of the flavonoids was assessed on *Staphylococcus aureus*. Bacteria exhibited sensitivity to apigenin, luteolin, morin and rutin at doses of 1 mM. Bacteriostatic effect was shown from quercetin at 0.05 mg/mL (1.6 × 10^−4^ M), 0.01 mg/mL (3.3 × 10^−5^ M), 0.5 mg/mL (1.6 × 10^−3^ M), 0.1 mg/mL (3.3 × 10^−4^ M), 1 mg/mL (3.3 × 10^−3^ M) and 10 mg/mL (3.3 × 10^−2^ M) and 30 mg/mL (9.9 × 10^−2^ M). Luteolin 1 mg/mL (3.4 × 10^−3^ M) and 5 mg/mL (1.7 × 10^−2^ M). Morin 1 mg/mL (3.3 × 10^−3^ M) and 10 mg/mL (3.3 × 10^−2^ M). Rutin 1 mg/mL (1.6 × 10^−3^M), catechin 1 mg/mL (3.4 × 10^−3^M) and 10 mg/mL (3.4 × 10^−2^ M), naringin 1 mg/mL (1.7 × 10^−3^ M), 10 mg/mL (1.7 × 10^−2^ M) and 30 mg/mL (5.1 × 10^−2^ M) (Table 2). For ^**g**^*S.a*. we found significant differences between rutin and luteolin with catechin and apigenin (*ρ* < 0.05).

### 3.6 Effect of flavonoids against *Agreggatibacter actinomycetemcomitans*

Effect from flavonoids against the anaerobic bacteria was shown from morin 10 mg/mL (3.3 × 10^−2^ M), quercetin 1 mg/mL (3.3 × 10^−3^ M),10 mg/mL (3.3 × 10^−2^ M), 30 mg/mL (9.9 × 10^−2^ M), catechin 10 mg/mL (3.4 × 10^−2^ M), luteolin 1 mg/mL (3.4 × 10^−3^ M), luteolin 5 mg/mL (1.7 × 10^−2^ M) and naringin 30 mg/mL (5.1 × 10^−2^ M) (Table 2). For ^a^*A.a.* there are significant differences between catechin, luteolin and naringin (*ρ* < 0.05).

### 3.7 Effect of flavonoids against *Streptococcous sanguinis and oralis*

There was not shown any antibacterial effect to these bacteria strains (Table 2). For *S.o.* and *S.s.* there is no significant difference between all of the flavonoids tested.

### 3.8 Effect of flavonoids against *Candida albicans*

Rutin and naringin exhibited inhibitory effect at doses of 100 μM; higher doses were required for morin (1 mM) to show inhibitory effect. An effect at 10 mg/mL (3.3 × 10^−2^ M) from Quercetin and Morin was shown to inhibit/stop *Candida albicans* growth. Luteolin inhibit *C. albicans* at 5 mg/mL (1.7 × 10^−2^ M) (Table 2). For ^**d**^*C.a.* we can observe significant differences between luteolin, rutin and naringin (*ρ* < 0.05).

## 4 Discussion

Data showed that luteolin, morin, naringin, quercetin and rutin inhibited *Actinomyces naeslundii, Actinomyces viscosus, Aggregatibacter actinomycecomitans, Enterococcus faecalis, Escherichia coli, Staphylococcus aureus and Lactobacillus casei* while apigenin showed activities against *Enterococcus faecalis* and *Staphylococcus aureus*. Catechin only had antibacterial activity against *A. actinomycecomitans* and *S. aureus*. We also found that luteolin, naringin, morin, quercetin and rutin inhibited *Candida albicans*. Morin had the most prevalent inhibition against 7 different strains of bacteria and *C. albicans.*

Pathogenic strains of species such as *Actinomyces, Escherichia coli, Staphylococcus aureus and Enterococcus faecalis* are known as multidrug resistant organisms and are a particular hazard to medical practice [30, 31]. Presently, no antibiotics are available for their treatment; for this reason, the development of new antibacterial drugs has become a priority. In recent years, several researchers have tried to identify edible, nontoxic compounds that could interfere with cariogenic biofilm formation. In this respect it has been demonstrated that certain proanthocyanidins obtained from cranberries may limit dental caries by inhibiting the synthesis of organic acids in *Streptococcus mutans* and *Streptococcus sobrinus* [32, 33]. Results of studies of proanthocyanidins on the ability of several oral species of *Streptococcus* to adhere to hydroxyapatite pellets pretreated with saliva showed that flavonoids significantly inhibited adhesion to the pellets [34]. Furthermore, hydrophobicity of the bacteria decreased with increasing flavonoid concentration. Recently, some studies reported that apigenin exerted activity against the streptococcal membrane by increasing their proton permeability and inhibition of acid production by *Streptococcus mutans* within biofilms [22, 35, 36, 37, 38]. Nevertheless, *Streptococcus mutans* is not the only organism causing caries. Dental caries etiology clearly points out its multi-factorial nature. Many other microorganisms with time, dietary factors and host factors promote caries. In the present study, we found that flavonoids exert activity against Gram-positive and Gram-negative microorganisms and against *Candida*. Recently, it has been shown that procyanidins disrupted acid production and acid tolerance of *Streptococcus mutans*. Further studies using individual cariogenic microorganisms shall elucidate the biological effects of flavonoids, they will advance our understanding on exact action mechanisms of these compounds. The susceptibility of certain strains of bacteria towards test compounds was evaluated by measuring inhibition diameter, from here it follows that increase of flavonoid concentration does not have any substantial influence on their antibacterial activity, this could be the result of a poor diffusion of these flavonoids in the agar plates.

However, molecule structure is closely related with their biological activity. Flavonoids tested in this study belong to polyhydroflavones. The molecules contain hydroxyl group substitutions at R3, R7 and R4’. Myricetin differs from the rest of flavonoids with a hydroxyl substitution on R5′, which represents just one hydroxyl group modification therefore has no antibacterial activity. Some researchers suggested that 2′, 4′-dihydroxylation of B ring and 5,7-dihydroxylation of the A ring are important for a significant inhibition of *Staphylococcus* growth.

In conclusion results presented in this research provide evidence that naturally occurring flavonoids exhibit antibacterial activity. Further research need to be conducted in order to establish the role of flavonoids in dental plaque control.

## Declarations

### Author contribution statement

Gloria Gutiérrez-Venegas: Conceived and designed the experiments; Performed the experiments; Analyzed and interpreted the data; Contributed reagents, materials, analysis tools or data; Wrote the paper.

Juan Arturo Gómez-Mora: Conceived and designed the experiments; Performed the experiments.

Marco Antonio Meraz-Rodríguez, Mónica Arisbet Flores-Sánchez: Conceived and designed the experiments; Performed the experiments; Analyzed and interpreted the data.

Laura Fabiola Ortiz-Miranda: Performed the experiments.

### Funding statement

The work was supported by grant from Dirección General de Asuntos del Personal Académico PAPIIT-201816.

### Competing interest statement

The authors declare no conflict of interest.

### Additional information

No additional information is available for this paper.
